# Mass spectrometric analysis of bioactive conditioned media of bacteria isolated from reptilian gut

**DOI:** 10.2144/fsoa-2023-0030

**Published:** 2023-05-02

**Authors:** Ruqaiyyah Siddiqui, Noor Akbar, Nelson Cruz Soares, Hamza Mohammad Al-Hroub, Mohammad Harb Semreen, Sutherland K Maciver, Naveed Ahmed Khan

**Affiliations:** 1 College of Arts & Sciences, American University of Sharjah, University City, Sharjah, 26666, United Arab Emirates; 2 Department of Medical Biology, Faculty of Medicine, Istinye University, Istanbul, 34010, Turkey; 3 Department of Clinical Sciences, College of Medicine, University of Sharjah, Sharjah, 27272, United Arab Emirates; 4 Department of Medicinal Chemistry, College of Pharmacy, University of Sharjah, Sharjah, 27272, United Arab Emirates; 5 Sharjah Institute for Medical Research, University of Sharjah, Sharjah, 27272, United Arab Emirates; 6 Centre for Discovery Brain Sciences, Edinburgh Medical School: Biomedical Sciences, University of Edinburgh, Edinburgh, UK

**Keywords:** antibiotic resistance, gut microbiota, metabolomics

## Abstract

**Aim:**

To determine whether selected gut bacteria of crocodile exhibit antibacterial properties.

**Materials & methods:**

Two bacteria isolated from *Crocodylus porosus* gut were used, namely: *Pseudomonas aeruginosa* and *Aeromonas dhakensis*. Conditioned media were tested against pathogenic bacteria and metabolites were analyzed using liquid chromatography-mass spectrometry.

**Results & conclusion:**

Antibacterial assays revealed that conditioned media showed potent effects against pathogenic Gram-positive and Gram-negative bacteria. LC–MS revealed identity of 210 metabolites. The abundant metabolites were, N-Acetyl-L-tyrosine, Acetaminophen, Trans-Ferulic acid, N, N-Dimethylformamide, Pyrocatechol, Cyclohexanone, Diphenhydramine, Melatonin, Gamma–terpinene, Cysteamine, 3-phenoxypropionic acid, Indole-3-carbinol, Benzaldehyde, Benzocaine, 2-Aminobenzoic acid, 3-Methylindole. These findings suggest that crocodile gut bacteria are potential source of novel bioactive molecules that can be utilized as pre/post/antibiotics for the benefit of human health.

## Plain language summary

Crocodiles thrive in unsanitary conditions, feed on rotten meat, and endure conditions that are detrimental to human health. In addition to their immune system, we speculate that their microbial gut flora produce substances contributing to their “hardiness” and “longevity”. Herein, we showed that selected bacteria isolated from crocodile gut produced potent antibacterial properties against multiple drug-resistant pathogenic Gram-negative and Gram-positive bacteria. LC–MS/MS revealed the identity of gut microbial metabolites. These findings suggest that analyses of crocodile gut bacteria may reveal potential drug leads that can be utilized as probiotics/pre/post/antibiotics for the benefit of human health, however intensive future research is needed to realize these expectations.

## Tweetable abstract

Gut microbiome of hardy crocodile is a potential pharmacy that should be exploited for human benefit.

## Graphical abstract

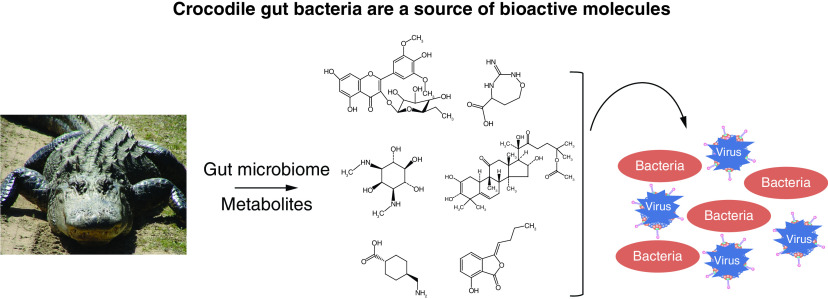

The advent of antibiotic resistance among pathogenic bacteria represents a foremost public health threat globally [1,2]. In particular, the rapid emergence and spread of antibiotic-resistant bacteria has become a global crisis, undermining antimicrobial discovery efforts and threatening the existence of *Homo sapiens* [3,4]. The number of antibiotic resistant infections are exceeding 2.8 million per year in the USA alone and continue to rise worldwide [5]. These findings highlight the need for the discovery of innovative antibacterial agents [6]. While we rely on novel antibacterial molecules to aid our ability to counter drug-resistant microbes, it is important to note that selected animal species thrive in polluted environment that are detrimental to human health. For example, cockroaches flourish in unhygienic conditions, while snakes eat germ-infested rats, and crocodiles can feed on rotten meat. How do such species ward-off disease and have existed over 300 million years inhabiting this planet, whereas *Homo sapiens* are threatened by diseases outbreaks? Moreover, these animals are constantly exposed to carcinogens and genotoxic heavy metals arsenic, cadmium, cobalt, chromium, lead, mercury, nickel, selenium, zinc and yet they have prolonged lifespan [7–10]. It is logical to study such species and their molecular mode of action for our benefit. Although the immune system of such animals plays the key role in their defense against disease-causing microbes, gut microbiota may also contribute to their ability to thwart infections. Moreover, previous studies have demonstrated that antimicrobial peptides and tissues from reptiles possess antibacterial activities [11,12], Although limited studies have focused on the antibacterial activities of the crocodile gut microbiota [13,14], there has been a recent study examining gut bacteria isolated from honey bees, against various bacterial strains such as *Bacillus subtilis* and *Morganella morganii*, which revealed anti-bacterial effects [15] and another study which investigated the role of diet in the gut microbiome of crocodile lizards [16].

Previous research as used bacteria/yeast of soil origin as a source for antibiotics but the bacteria of GI tract of animals inhabiting unhygienic environments offer an important bacterial flora that ought to be explored for potentially novel antimicrobials [10]. Microbes have remained a valuable resource for early-stage antibiotic discovery, producing innumerable useful secondary metabolites and other bioactive compounds [17]. To overcome competition, these compounds are produced as biological weapons against other microbes in a complex microbial community [18]. The producer organism is not affected by these metabolites. Several classes of compounds have been isolated from microbes which affect biological activities including antimicrobial properties [19–21]. For example, *Bacillus subtilis* produce antimicrobial peptides exhibiting broad-spectrum antibacterial and antifungal activities [22]. Soil microorganisms, particularly bacteria, produces a plethora of secondary metabolites, some of which can inhibit/kill other microbes in competition [23]. Symbiotic bacteria from marine environment showed exceptional antibacterial activities against *Bacillus subtilis*, *Mycobacterium smegmatis* and *Staphylococcus aureus*, *E. coli*, *Salmonella enterica* and *Pseudomonas aeruginosa* [24]. Similarly, *Bacillus* species isolated from sponges produced potential antibacterial metabolites [25]. Bacteria isolated from marine sponges produced alkaloids, flavonyl glycosides, quinones, and flavonoids having tremendous antibacterial activity [26]. In addition, several reports have indicated that *Pseudomonas* strains produce substances exhibiting antibacterial activities [27–29].

In most cases, marketed antibiotics have been derived from fungi/bacteria isolated from the environment, we speculate that bacteria inhabiting animals gut can also prove to be useful source of innovative antibacterials. Herein, we evaluated bacteria crocodile (*Crocodylus porosus*) gut. Bacteria were selected based on their potent metabolic activities [14] and their conditioned media (CM) were evaluated against a panel of Gram-positive and Gram-negative MDR clinical isolates. These metabolites /extracts were subjected to high through put LC–MS/MS for their characterization and molecular identity.

## Materials & methods

### Bacteria cultivation

Numerous bacteria were utilized in the current study as detailed in Table 1.

**Table 1.** Bacteria used in this study.

Bacteria	Strain
*Bacillus cereus*	MTCC 131621 (clinical isolate)
*Streptococcus pneumoniae*	ATCC 13883 (clinical isolate)
*Streptococcus pyogenes*	ATCC 49399 (clinical isolate)
Methicillin-resistant *Staphylococcus aureus*	MTCC 381123 (clinical isolate)
*Escherichia coli* K1	MTCC 710859 (clinical isolate)
*Pseudomonas aeruginosa*	ATCC 10145 (clinical isolate)
*Klebsiella pneumoniae*	ATCC 13883 (clinical isolate)
*Serratia marcescens*	MTTC 13880 (clinical isolate)
*Salmonella enterica*	ATTC 14028 (clinical isolate)

### Crocodile gut bacteria

Isolation and identification of crocodile gut bacteria was accomplished as described previously [14]. In brief, department of Wildlife and National Parks (PERHILITAN), Malaysia, approved this study, as well as Sunway University, Malaysia (SUNREC 2019/023). Additionally, we also confirmed that all the experiments were carried out in agreement with appropriate protocols and guidelines as formerly defined [14]. The gut bacteria were isolated using sterile cotton swabs [14]. Bacteria were streaked on blood agar plates. Several bacteria were observed and further differentiated based on their appearance. Finally, bacterial identification was accomplished using microbiological as well as 16S rRNA gene amplification and sequencing as described previously [14]. Among several bacteria, two bacteria isolated from crocodile gut were used, namely: *P. aeruginosa* (CM1) and *A. dhakensis* (CM2).

### Bacterial conditioned medium preparation

Preparation of conditioned media were accomplished as described previously [14]. In brief, axenic bacteria were cultured in RPMI-1640 at 37°C for 48 h with continuous shaking [14,20,30]. Next, grown bacterial cultures were centrifuged at 4°C for 1 h at 10,000 × *g*. Last, culture supernatants were filter-sterilized (0.22 μm pore size filter) and prepared CM were kept at -80°C.

### Antibacterial assays

To determine CM effects against bacteria, antibacterial assays were carried out as described earlier [20,31]. In brief, different bacteria, as indicated in Table 1, were grown overnight and cultures were adjusted to optical density (OD) of 0.22 at 595 nm using a spectrophotometer (OD_595_ = 0.22) which corresponds to approximately 10^8^ colony-forming units (CFU) per ml. Next, an inoculum of 10 μl of above bacterial culture (~1 × 10^6^ CFU) were challenged with 100 μl of bacterial CM at 37°C for 120 min. Following this incubation, tenfold serial dilution was done, and 10 μl of each dilution was plated on nutrient agar plates. Next, plates were incubated at 37°C for overnight and the number of bacterial colonies were enumerated. Bacteria grown in phosphate buffer saline (PBS) or incubated with non-pathogenic *E. coli* K-12 CM, or with gentamicin (100 μg/ml) were used as controls.

### Ultra-performance liquid chromatography tandem mass spectrometry (HPLC–MS/MS)

The CM were analyzed by Q-TOF MS as described previously [32]. Briefly, samples were separated using Elute HPG 1300 pumps and Elute Autosampler. For metabolomics, 10 μl was injected twice for each sample and eluted using a 30-min gradient with flow rates of 250 μl/min for elution and 350 μl/min for re-equilibration, followed by analysis using TimsTOF with Apollo II electrospray ionization (ESI) source. MetaboScape^®^ 4.0 was used for statistical analysis and metabolite processing. Bucketing parameters for molecular feature detection of the processed data in T-ReX 2D/3D workflow were as follows: intensity threshold equal to 1000 counts along with minimum peak length of 7 spectra; utilizing peak area for quantifying the feature. The parameters for data bucketing were assigned as follows: Retention time range started at 0.3 min and ended at 25 min, while mass range started at 50 m/z and ended at 1300 m/z with features ranging in at least 3 of 18 samples, and the MS/MS import method was done using the average spectrum out of all MS/MS spectra. Identification of metabolites was based on mapping the MS/MS spectra and retention time in the HMDB 4.0, an annotated resource designed to satisfy the needs of the metabolomics community. The compounds with MS/MS were identified using library matching through the annotation process. Then, the selected metabolites were filtered by choosing the set with a higher annotation quality score (AQ score) representing the best retention time values, MS/MS score, m/z values, mSigma, and analyte list spectral library. The threshold for significance was denoted with p < 0.05. Gene Ontology (GO) term and pathway enrichment analyses were accomplished using metaboanalyst (metaboanalyst.ca) using *Pseudomonas putida* KT2440 database. All data, including the raw QGD files, have been deposited in the Metabolomics Workbench repository (metabolomicsworkbench.org) and currently under process. Alternately, the obtained spectra were processed using MS-DIAL (http://prime.psc.riken.jp/compms/msdial/main.html) in this instance, and all features having MS2 spectra were identified using a search against the databases for natural products, notably COCONUT, UNPDB, NPA and KNApSACK. All assignments that either had no hits or hits to HMBD were filtered out.

## Results

### Bacteria isolated from crocodile gut

In our previous work, several bacteria were isolated from crocodile gut as indicated prior (Khan *et al.*, 2021) and these bacteria were grown and CM was prepared. Bacterial CM with the most potent activities were selected based on experimentation conducted in our previous study, which investigated cell metabolic activity via the MTT 3-(4,5-dimethylthiazol-2-yl)-2,5-diphenyltetrazolium bromide assays and cell survival assays [14]. Based on earlier study, two bacteria exhibited potent activity, hence selected for this study. These included: *P. aeruginosa* and *Aeromonas dhakensis*, as they depicted the most potent inhibition of cell metabolic activity or viability reduction, and cell survival inhibition in cancer cell lines, previously [14]. Their CM were prepared as discussed in methodology section. Last, these CM were evaluated against Gram-positive and Gram-negative MDR clinical isolates (Table 1). Of note, *P. aeruginosa* is referred to as CM1, while *A. dhakensis* is referred to as CM2 throughout the manuscript.

### Bacterial conditioned media isolated from crocodile gut presented antibacterial effects

**Figure 1.**
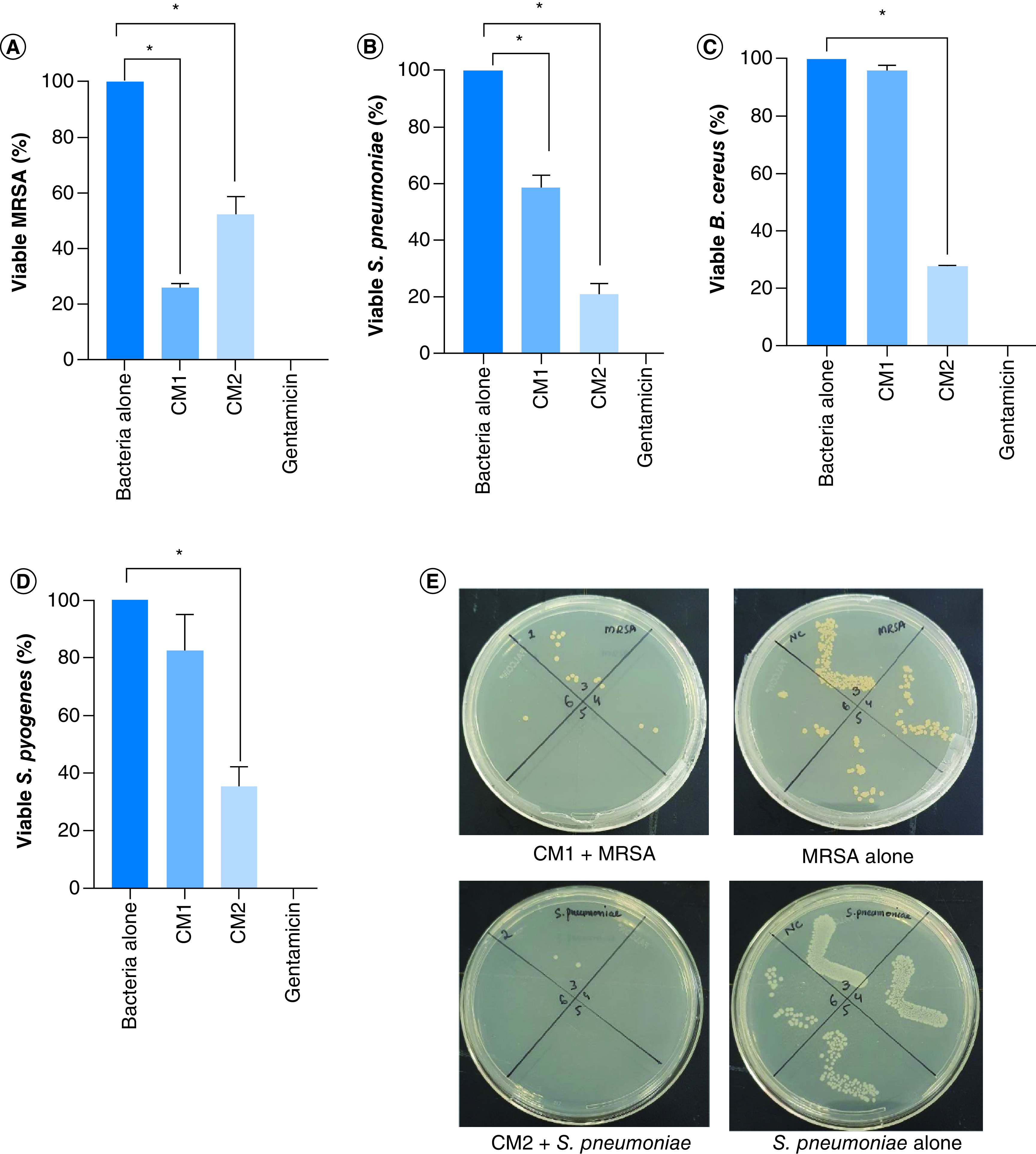
Crocodile gut bacterial conditioned media displayed potent bactericidal activity versus Gram-positive bacteria. **(A)** CM tested against MRSA, **(B)** *S. pneumoniae* **(C)** *B. cereus* and **(D)** *S. pyogenes*. **(E)** Representative effects of CM against MRSA and *S. pneumoniae*. Data presented here comprise the mean ± standard error of three-times independent experiments accomplished in duplicates. p-values were elucidated using student's *t*-test. *p ≤ 0.05. CM1: *P. aeruginosa*; CM2: *A. dhakensis*; MRSA: Methicillin-resistant *Staphylococcus aureus*.

Bacterial extracts (conditioned media) were assessed against a range of MDR bacteria. The results showed that both CM showed bactericidal effects against methicillin-resistant *Staphylococcus aureus* (MRSA) and *S. pneumoniae* (using student's *t*-test, two-tailed distribution, p < 0.05) (Figure 1A, B & Table 2). Similarly, when tested against *B. cereus* and *S.* pyogenes, only CM2 showed notable antibacterial activities while CM1 did not show the effect (p < 0.05) (Figure 1C, D & Table 2). CM from K12 did not reveal any antibacterial effects (data not shown).

**Table 2.** Representation of bactericidal effects of conditioned media against Gram-negative and Gram-positive clinical isolates.

Conditioned media	Bactericidal effects vs Gram +ve bacteria				Bactericidal activities vs Gram –ve bacteria				
	*B. cereus*	MRSA	*S. pyogenes*	*S. pneumonia*	*E. coli*	*K. pneumoniae*	*P. aeruginosa*	*S. marcescens*	*S. enterica*
CM1	-	+	-	+	+	-	+	-	+
CM2	+	+	+	+	-	-	+	+	+

CM1: *P. aeruginosa*; CM2: *A. dhakensis*; MRSA: Methicillin-resistant *Staphylococcus aureus*.

**Figure 2.**
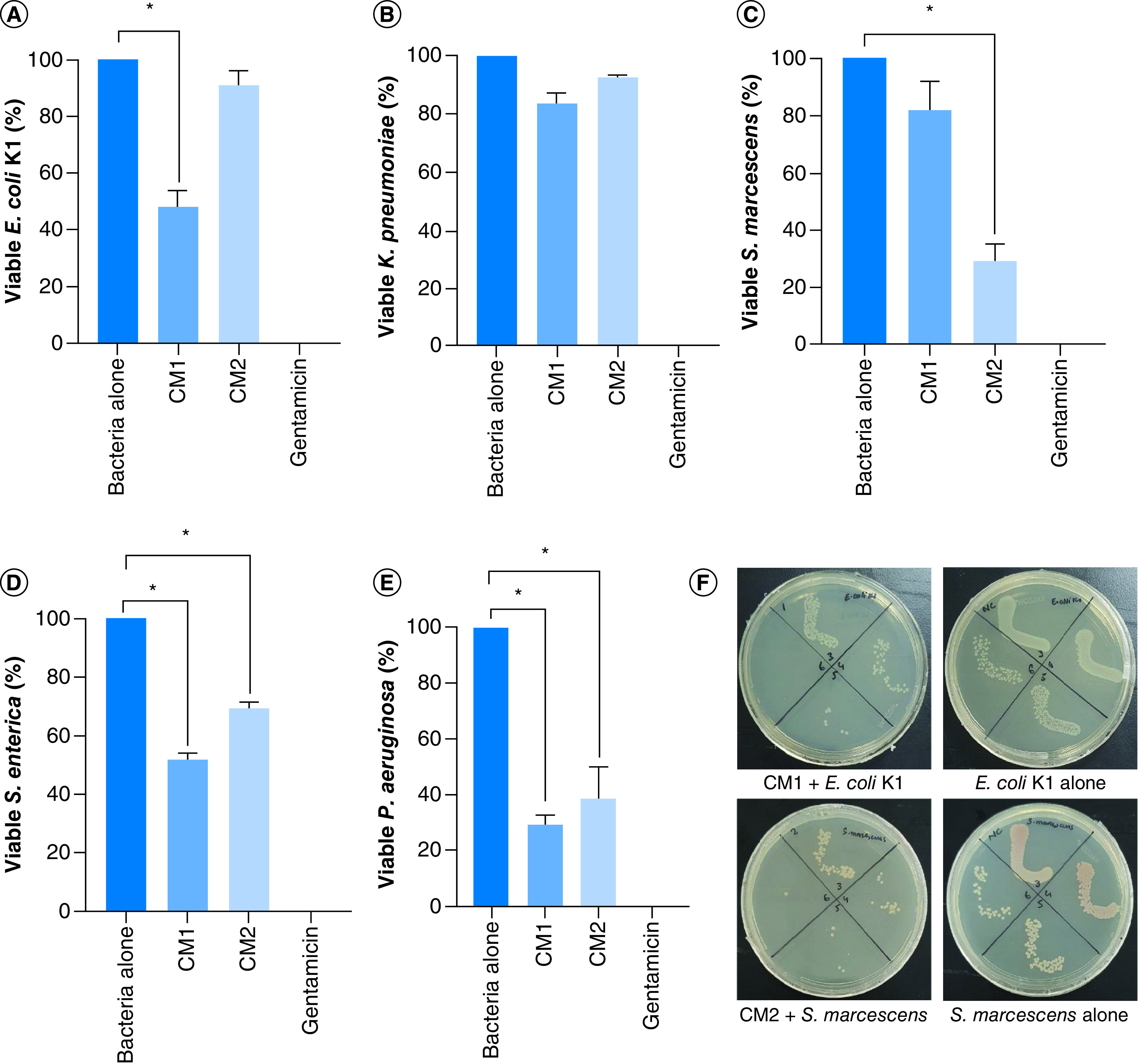
CM from crocodile bacteria revealed bactericidal effects. The data are expressed as the mean ± standard error of three independent experiments accomplished in duplicates. *P*-values were elucidated using two sample *T*-test. (*) denotes *P* ≤ 0.05. **(A)** when the CM were tested against *E. coli* K1, **(B)** against *K. pneumoniae*, **(C)** against *S. marcescens* **(D)** versus *S. enterica* and **(E)** against *P. aeruginosa*. **(F)** Demonstrative effects of CM against *E. coli* K1 and *S. marcescens*. CM1: *P. aeruginosa*; CM2: *A. dhakensis*.

Correspondingly, the results indicate that CM1 showed important antibacterial effects against *E. coli* K1 (p < 0.05) (Figure 2A & Table 2) while CM2 failed to show the activity. Against *K. pneumoniae*, both the CM i.e., CM1 and CM2 did not show any effects (p < 0.05) (Figure 2B & Table 2). When CM were evaluated versus *S. marcescens*, only CM2 showed remarkable bactericidal activity (p < 0.05) (Figure 2C & Table 2). Finally, both the CM exhibited significant antibacterial properties against *S. enterica* and *P. aeruginosa* (p < 0.05) (Figure 2D, E & Table 2).

### Mass spectrometry revealed several secondary metabolites

**Figure 3.**
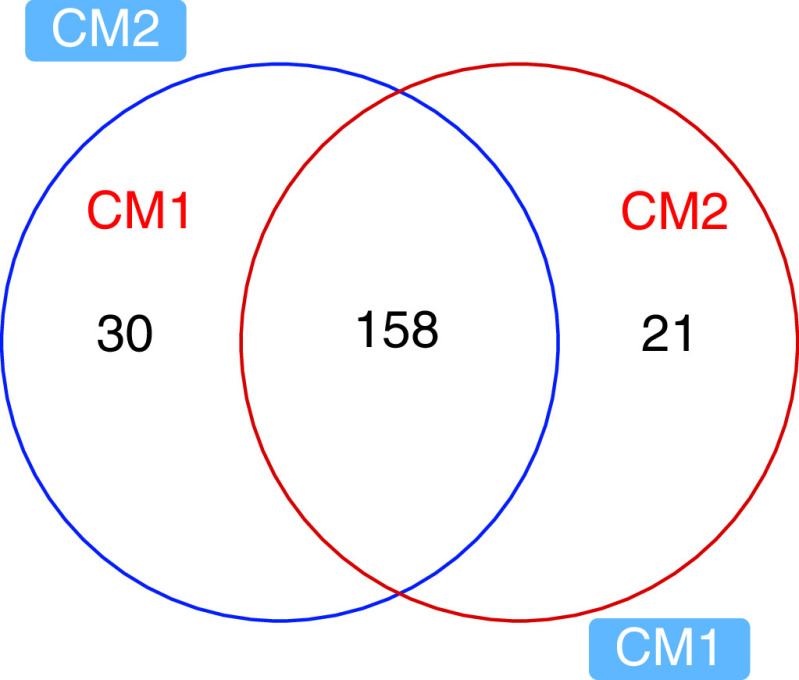
C Venn diagram showing metabolites common and unique between CM1 and CM2. CM1: *P. aeruginosa*; *CM2: A. dhakensis*.

**Figure 4.**
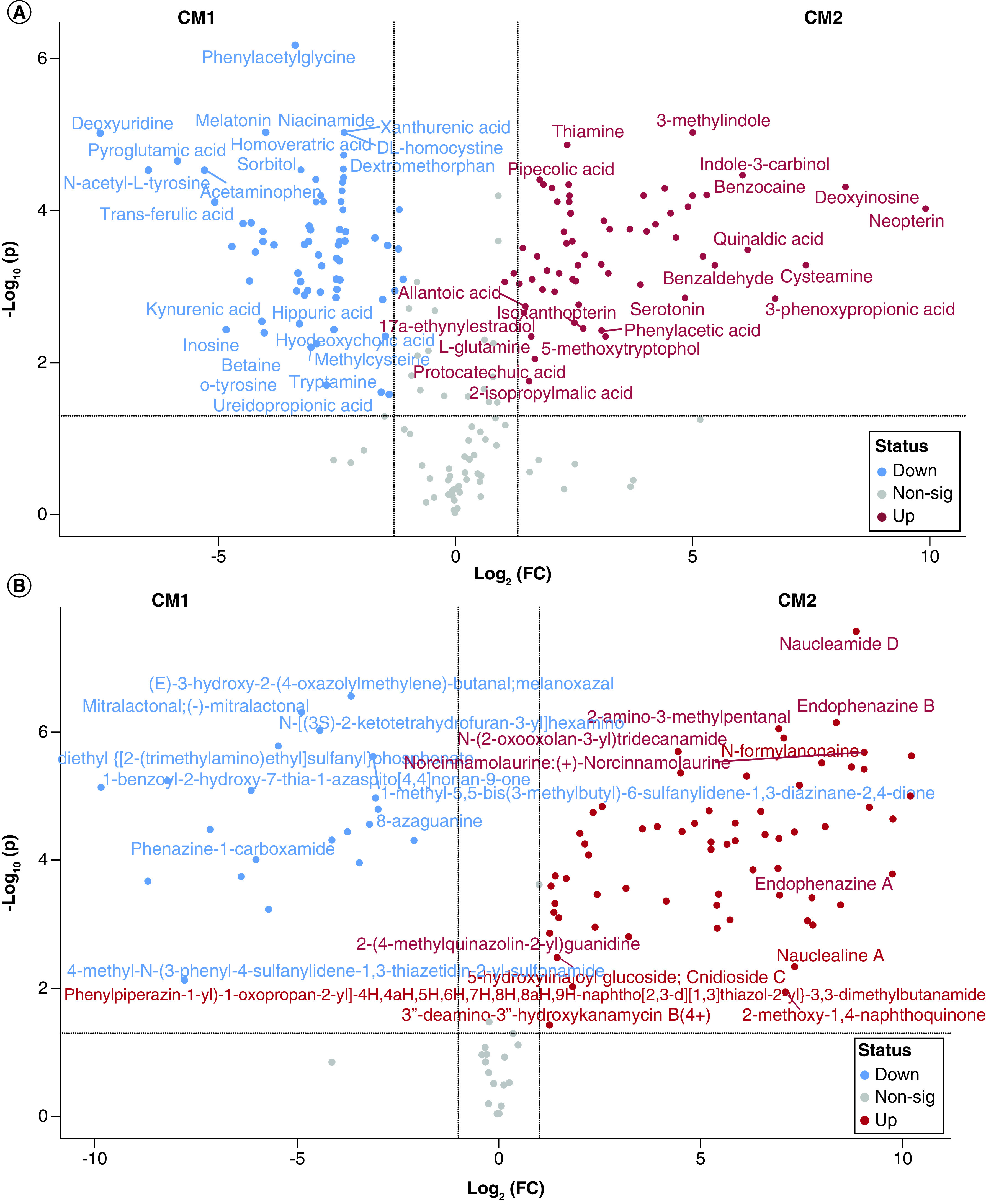
Metabolites produced by crocodile gut bacteria, i.e., CM1 (*P. aeruginosa*) and CM2 (*A. dhakensis*). **(A)** Volcano plot showing those metabolites identified using HMBD database with significantly altered abundance in CM1 and CM2. Scatter plot of (log2-transformed) fold-change versus significance, highlighting those metabolites with fold-change greater than 2 and false discover rate- (FDR) adjusted p-values <0.05 (-log10(p-values) >1.3). **(B)** Volcano plot showing those metabolites identified using Natural Compounds database with significantly altered abundance in CM1 and CM2. Scatter plot of (log2-transformed) fold-change versus significance, highlighting those metabolites with fold-change greater than 2 and false discover rate- (FDR) adjusted p-values <0.05 (-log10(p-values) >1.3). CM1: *P. aeruginosa*; CM2: *A. dhakensis*.

The analyses resulted in 210 highly confidently (MS/MS) identified metabolites in both sample samples CM1 and CM2 using the HMBD database (Supplementary Table 1). The pairwise comparison of the two samples indicated that 137 metabolites change significantly between them (p < 0.05), with 74 and 63 were more abundant in CM2 and CM1, respectively. Among abundant metabolites were, N-Acetyl-L-tyrosine, Acetaminophen, Trans-Ferulic acid, N,N-Dimethylformamide, Pyrocatechol, Cyclohexanone, Diphenhydramine, Melatonin, Gamma-terpinene, Cysteamine,, 3-phenoxypropionic acid, Indole-3-carbinol, Benzaldehyde, Benzocaine, 2-Aminobenzoic acid, 3-Methylindole (Figures 3, 4A & Supplementary Table 1).

**Figure 5.**
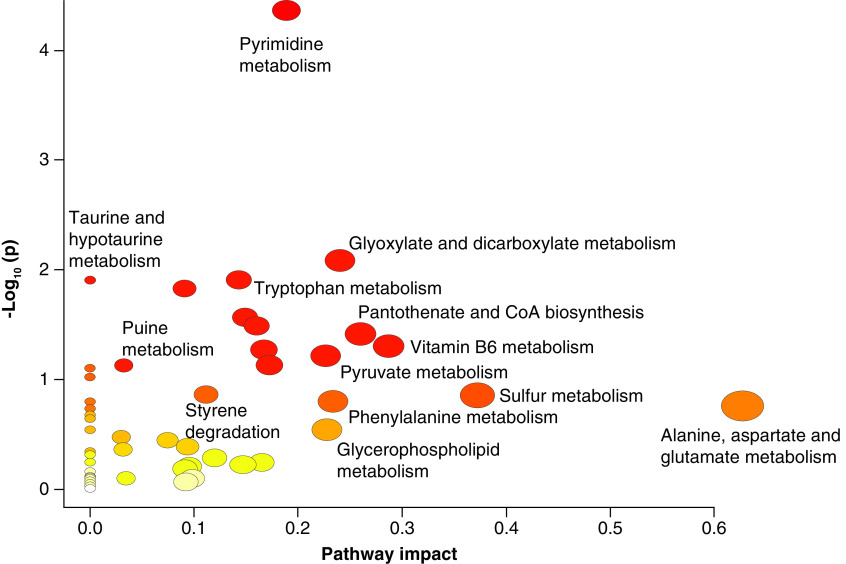
Pathway enrichment analysis using MetaboAnalyst of the metabolites that altered significantly in CM1 and CM2. Visualization of Joint pathway enrichment analyses. Nodes are colored according to –log10(p) and sized according to the number of associated members metabolites. p < 0.05.

Pathway enrichment of the metabolites sets based on bacterial pathways, showed that they were enriched for top ten pathways associated with the vitamin B6 metabolism, taurine and hypotaurine metabolism, styrene degradation, sulfur metabolism, glyoxylate and diacarboxylate, tryptophan metabolism, pantothenate and CoA biosynthesis (Figure 5 & Table 3).

**Table 3.** Summary of the most abundant metabolites in both samples CM1 and CM2 that were identified using HMBD database.

Metabolite	CM2 (intensity)	CM1 (intensity)
Phenylacetylglycine	333886	32560
Phosphoric acid	199618	327548
N,N-Demethylfromamide	115058	4970
Acetic acid	109014	113966
Elaidic acid	96640	73090
1,3,5-Trimethoxybenzene	83202	307206
Tryptophanol	83202	307206
Alpha-N-phenylacetyl-L-glutamine	65984	80174
Diphenhydramine	64394	3644
6-Dimethylaminopurine	62592	57934
Propranolol	59548	8340
Hyodeoxycholic acid	53672	5948
L-Phenylalanine	53408	4338
Trans-Ferulic acid	49788	1430
Melatonin	48512	2968
Metabolite	CM2 (intensity)	CM1 (intensity)
Indole-3-carbinol	9284	681338
5-Hydroxytryptophol	14102	215332
2,3-Diaminopropionic acid	5944	154404
Pipecolic acid	42310	144144
Alpha-N-phenylacetyl-L-glutamine	65984	80174
Sulfite	16496	73762
3-Methylindole	2634	71182
6-Dimethylaminopurine	62592	57934
Isobutyric acid	41920	55830
Deoxyinosine	248	54152
Benzocaine	1272	51118

When searched against the natural compound database (see materials and methods) 110 metabolites were identified in both CM1 and CM2 samples. Of these 87 were significantly abundant between the analyzed samples and 28 compounds had previously associated with a bioactivity including antibiotic, antibacterial, anticoccidial, antifungal, anticancer, anti-inflammatory and others as summarized in Table 4 & Figure 4B.

**Table 4.** Summary of the most abundant metabolites in both samples CM1 and CM2 that were identified using natural database.

Metabolite	CM1 (intensity)	CM2 (intensity)	Bioactivity
Arohynapene D	2765	3269	Anticoccidial agent
2-(4-methylquinazolin-2-yl)guanidine	1285	3271	Myeloperoxidase inhibitor
phenazine-1-carboxamide	30576	452	antifungal
2-[4′-(Methylamino)phenyl]quinazolin-4(3H)-one	2422	2644	Luciferase inhibitor
Nannozinone B	2510	3307	Antibacterial and antifungal activity
(+)-Thienamycin;Thienamycin	2664	1498	New antibiotics
Solanapyrone A	1988	2723	Antibiotic activity
Hynapene A	27594	2968	Anticoccidial agent
Endophenazine A	86	82758	Antibiotic
Endophenazine B	160	52717	Antibiotic
Naucleofficine D;(-)-Naucleofficine D	53	9647	Antibacterial activity
Sennecicannabine	72	2436	Antileishmanial compound

## Discussion

For evolutionary success in the microbial world, a crucial prerequisite is the ability to endure and divide in the surrounding and often overwhelming microbial species [8–10]. To cope with such distressing conditions, microorganisms produce and secrete countless secondary metabolites to inhibit other species and survive and reproduce in milieus [33]. Furthermore, previously we investigated the inhibitory effects of crocodile gut bacteria against cancerous cell lineages and determined their metabolic activities [14]. However, the objective of the current study was to determine whether bacteria of crocodile produce bioactive molecules with potential antimicrobial activities. Two bacterial species were tested for antibacterial activities for potential use as bioactive molecules was examined. Potent antimicrobial activities were observed versus various MDR bacteria such as MRSA.

Previously, earlier findings depicted that gut bacteria of animals such as reptiles possess antimicrobial activities. For example, previously we isolated the gut bacteria from the sea turtle (*Cuora amboinensis*) which displayed potent antibacterial molecules as well as minimal cytotoxicity against human cell lines. In this study, *P. aeruginosa* produced a wide range of secondary metabolites including 4-hydroxy-2-alkylquinolines, rhamnolipids and novel *N-* acylhomoserine lactone [30]. Similarly, cockroach gut bacteria produce several bioactive metabolites that showed remarkable bactericidal effects against Gram-negative (*E. coli* K1, *P. aeruginosa, S. marcescens, S. enterica* and *K. pneumoniae*) and Gram-positive (MRSA, *B. cereus* and *S. pyogenes*) bacteria [20]. Other studies isolated bacteria from grasshopper's gut that exhibited antibacterial activities against *E. coli* and other fugal pathogens [34]. Springtail gut bacteria were isolated that showed significant antibacterial and antifungal activities [35]. In human gut, the commensal microbiota helps in regulating gut health by producing valuable metabolites that help the host immune system in the elimination of pathogenic microbes [36]. Similarly, bacteria present in the insect gut produces bioactive molecules that stimulate the insect immunity against the pathogenic invaders [37]. All these examples show that bacteria isolated from interesting species may be a source of potential innovative resource for antibacterial agents [10].

Among the metabolites identified in our study, we observed that several metabolites have been associated previously with gut microbiota of humans and other animals such as L-tryptophanol, Urocanic acid, allantoic acid, and Isovalerylcarnitine [14]. Interestingly, the metabolomic analysis revealed several metabolites associated with antimicrobial agents some with antibiofilm activity such as cysteamine, sisomicin with a known action against biofilms and with an antiprotozoan action. It is likely that the microbiota is composed of several bacterial species that control not only the proliferation of other harmful microbes such as protozoa, but also regulate the pathogenicity, including *P. aeruginosa*, by preventing biofilm formation, in order to prevent an imbalanced microbiota. Interestingly, the meta-proteomic dataset and metabolomics analysis indicated that the isolated bacterial species have preserved metabolic markers that may be linked to their natural habitat and close interaction with the host.

Of note, the list of the metabolites from our study included a number of anti-inflammatory metabolites 5-methoxytryptophan, and 3,4-Dihroxyphenylglycol. Furthermore, several other metabolites associated were associated with anticancer action, such as nifedipine, 4-chlorotestosterone-17-acetate, 3-phenoxypropionic acid. In comparison, a previous study which identified metabolites from *Pseudomonas aeruginosa* associated with an Antarctic sponge, revealed the presence of series of diketopiperazines and two phenazine alkaloid antibiotics [38]. Further, another study investigating the novel metabolites *Pseudomonas kilonensis* revealed Methyl diethanol amine, Aspartylglycine ethyl ester, Phosphonothioic acid propyl-O, S-dimethyl ester, O, O, O-Trimethyl thiophosphate and omethoate, which appear to differ from the metabolites observed in our study [39]. Similarly, metabolite analysis of *aeromonas* culture media revealed the metabolites :9-chlorolumichrome, veronimide and veronipyrazine, and several known diketopiperazines, suggestive of the unique properties of each bacterial strain [40]. Further work encoding the genes and examining the biosynthetic pathways of the potential antibiotics produced in the CM from crocodile gut microbiota need to be determined, for example bacterial strains of *Pseudomonas* are known for their ecological and metabolic diversity [41].

Based on the presence of the various metabolites in the crocodile bacterial CM studied herein, it is likely that these metabolites act together in complex interactions, and may be providing their host, i.e. the crocodile with beneficial capabilities in order to thrive and prosper in harsh environments, albeit the precise details need to be studied further. It is tempting to speculate that the crocodile gut microbiota may explain in part why these animals are noted for their hardiness and not suffering from intestinal inflammatory disorders such as cancer. One of the limitations of this study was that we focused on aerobic culturable bacteria and future studies are needed to determine anaerobic bacteria and other unculturable bacteria/other microbes that may also be a potential source of novel metabolites protecting the crocodile from adverse conditions. Consequently, these microbes should also be recovered and the activities of their metabolites assessed in the future studies, *in vitro* and *in vivo*, as well as determine their molecular mechanisms of action. Future studies will also test other clinical and environmental strains of *A. dhakensis* and *P. aeruginosa* for comparative metabolomics. If other *Pseudomonas* or *Aeromonas* strains CMs exhibit dissimilar antibacterial activity, then it is related to the crocodile gut, otherwise it could be strain related. This remains a limitation of the study and will be investigated in the future study. The present study involved bacteria isolated from a crocodile and data obtained from subsequent experiments conducted, are hypothesis-confirming experiments and further molecular studies are needed to determine their potential translational value. Likewise, the mechanism of action of the conditioned media containing bacterial metabolites needs to be investigated. Based on the most abundant metabolites present, such as Arohynapene D and Hynapene A, which are known to be antimicrobial agents, and Nannozinone B, Solanapyrone A, Endophenazine A are known to have antibacterial activities, whereas 2-[4′-(Methylamino)phenyl]quinazolin-4(3H)-one is a luciferase inhibitor, among several others, alone or in combination are of interest. Nonetheless, in depth mechanistic molecular studies are needed to determine the potential translational value of these findings.

Moreover, bacterial conditioned media were prepared using RPMI-1640, and the composition of RPMI-1640 include several different compounds (such as: aspartic acid; glutamic acid; asparagine; serine; glutamine; histidine; glycine; threonine; arginine; alanine; tyrosine; cystine; valine; methionine; norvaline; tryptophan; phenylalanine; isoleucine; leucine; lysine) which could interfere with our findings, hence, our results should be interpreted in view of RPMI composition. Nonetheless, in depth analysis and characterization of these innovative metabolites is warranted and these should be tested further *in vitro* and *in vivo* as well as animal models with a defined microbiota such as gnotobiotic mice [10]. This will facilitate comprehension of their precise role, interaction with each other as well as with the immune system and assess their prospective use as pre/post/antibiotics for the benefit of human health.

## Conclusion

Two bacteria isolated from crocodile gut produced a plethora of metabolites that showed potent antibacterial effects. Based on these findings, it is tempting to speculate that the crocodile gut microbiota may explain in part why these animals are noted for their hardiness and withstand exposure to conditions that are detrimental to human health. Further analyses of crocodile gut bacteria may reveal potential drug leads, however intensive future research is needed to realize these expectations.

**Summary points**

- Species such as Crocodile are able to ward-off disease despite exposure to unhygienic conditions, suggesting that such species must have developed mechanisms to protect themselves from pathogens. Besides their immunity to fight pathogens, we hypothesize that their microbial gut flora produces bioactive molecules to thwart infections. The aim of this study is to source microbes inhabiting unusual environmental niches such as crocodile gut for their antibacterial properties.
- Selected bacteria isolated from the crocodile gut were used and their conditioned media were prepared. Assays revealed that crocodile gut bacteria exhibit remarkable antibacterial activity against a panel of multiple drug-resistant Gram-negative and Gram-positive bacteria.
- Bioassay-guided testing of selected bacterial conditioned media using LC-TIMS-QTOF MS, revealed the identity of 210 metabolites including N-Acetyl-L-tyrosine, Acetaminophen, Trans-Ferulic acid, N, N-Dimethylformamide, Pyrocatechol, Cyclohexanone, Diphenhydramine, Melatonin, Gamma–terpinene, Cysteamine, 3-phenoxypropionic acid, Indole-3-carbinol, Benzaldehyde, Benzocaine, 2-Aminobenzoic acid, 3-Methylindole etc.
- Overall, our findings suggest that analyses of crocodile gut bacteria may reveal potential drug leads, and we ought to learn from such species and determine their protective mechanisms and/or isolate bioactive molecules and use to our advantage as pre/pro-post-biotics. Because we are exploring untapped sources, it is believed that several bioactive molecules have potential to counter human disorders.
